# Worry Intervention in an Older Adult With a Persecutory Delusion: A Single Case Experimental Design

**DOI:** 10.32872/cpe.11173

**Published:** 2023-09-29

**Authors:** Poppy Brown, Anna Crabtree

**Affiliations:** 1 Oxford Institute for Clinical Psychology Training and Research, Oxford Health NHS Foundation trust and University of Oxford, Warneford Hospital, Oxford, United Kingdom; 2 Royal Holloway University of London, Egham Hill, Egham, United Kingdom; University of Hamburg, Hamburg, Germany

**Keywords:** worry, paranoia, persecutory delusion, older adult, single case experimental design

## Abstract

**Background:**

This report presents the single case of Jack, a 67-year-old referred to our Older Adult Community Mental Health Team (OA CMHT) for his distressing persecutory delusion and high levels of worry. Jack also reported learning difficulties and autistic traits, although neither were formally diagnosed.

**Method:**

Ten sessions of worry intervention taken from The Feeling Safe Programme worry module were used to reduce Jack’s time spent worrying and increase his engagement in meaningful activity. Weekly face-to-face sessions were held, with Jack’s brother acting as a co-therapist. Adaptations to the intervention were made based on Jack’s learning preferences. An AB single case experimental design was adopted to compare Jack’s scores on measures of worry, paranoia and delusional conviction, and wellbeing and daily functioning before and after intervention.

**Results:**

Results demonstrate the worry intervention improved Jack’s scores on all measures to a clinically significant degree.

**Conclusions:**

This is the first known report of applying the worry intervention to an older adult. The results show the intervention can be of considerable benefit in terms of reducing worry and paranoia, in the context of both older age and suspected neurodiversity.

## Highlights

- The evidence base for treating persecutory delusions through CBT is growing, but we do not know if these treatments are acceptable to, and effective with, older adults.
- This is the first known use of the worry module from The Feeling Safe Programme with an older adult.
- Significant reductions in paranoia and worry and improvements in wellbeing and functioning were seen.
- The intervention is brief and can be adapted to the learning needs of the individual.

## Overview of the Literature

A persecutory delusion is a severe form of paranoia, where an individual holds a distressing belief with high conviction (above 50% certainty) about being at risk of harm from others (Freeman, 2016). Persecutory delusions are one of the most common symptoms of psychosis and can have a severe impact on an individual’s life and wellbeing. Half of those experiencing persecutory delusions report levels of psychological wellbeing in the lowest 2% of the general population (Freeman et al., 2014). Levels of anxious avoidance are often comparable to what is seen in agoraphobia, and levels of worry are comparable to generalised anxiety disorder (Freeman, Taylor, et al., 2019).

A significant number of older adults are likely to be living with psychosis, including persecutory delusions, likely due to a combination of these symptoms being persistent over many years from first onset, and an estimated 2% of individuals experiencing a first episode of psychosis after the age of 65 (excluding psychotic experiences in the context of dementia) (Mitford et al., 2010; Vasiliadis et al., 2022). Despite this, there is limited research looking at persecutory delusions in older adults and no separate NICE guidance for treating psychosis in older adults. Older adults also do not have the same access to Assertive Outreach, Crisis, and Early Intervention in Psychosis (EIP) teams as working age adults. EIP services, for example, typically only accept referrals of patients aged 15-65, despite guidance stating that these services do not exclude individuals based on their age (Royal College of Psychiatrists, 2018).

NICE guidance for treating psychosis in adults recommends oral anti-psychotic medication in conjunction with psychological intervention – family intervention or CBT. A recent meta-analysis concluded that CBT was more effective for both hallucinations and delusions when compared with any control (Turner et al., 2020), and that the evidence base for its effectiveness is robust. Furthermore, a number of randomised controlled trials tailoring aspects of CBT to treat persecutory delusions have shown very positive effects; for example, The Worry Intervention Trial (Freeman et al., 2015). Compared with standard care, this eight-week worry intervention significantly reduced levels of worry and paranoia in 150 patients with persecutory delusions, adding to evidence that worry is a causal factor in the development and persistence of persecutory delusions that can be successfully ameliorated through intervention. The Worry Intervention now forms one of six modules within the Feeling Safe Programme, a modular psychological therapy for persecutory delusions that targets mechanisms (such as worry), that are known to cause and maintain paranoia (Freeman, Emsley, et al., 2021). The Feeling Safe Programme as a whole has demonstrated effect size improvements in delusions far above any previous intervention (Cohen’s *d* = 1.2 versus Cohen’s *d* = 0.3 for generic CBT for psychosis; Bighelli et al., 2018; van der Gaag et al., 2014) and training on this intervention is now being delivered to clinicians across England. In the recent trial of the intervention, patients typically completed two-to-three of the six modules in total, with the worry intervention most commonly being delivered first.

Given our ageing population, the number of older adults living with psychosis is likely to increase. This could have major clinical, social, and economic implications (Mitford et al., 2010). Evidence suggests older adults typically have more positive help-seeking attitudes than younger adults, meaning their low use of mental health services may be better explained by lack of service provision and/or ageism within healthcare in some cases (Mackenzie et al., 2008). To reduce ageism, provision must be based on need and appropriateness, rather than age. It is therefore important to assess whether current treatments used with adults of working age are also acceptable to, and effective, with older adults, with consideration given to the unique difficulties that older adults may more commonly face. This may include poor physical health, bereavement, and changes in roles e.g. from carer to being cared for, given there is evidence to suggest these factors can negatively impact mental health and therefore need to be considered in older adult’s formulations (Laidlaw et al., 2004, 2016). Only one older adult was recruited in the Feeling Safe Trial, and older adults were excluded entirely from the Worry Intervention Trial. This report therefore presents the first known case of applying the worry intervention to an older adult with a persecutory delusion.

## Introduction to the Case

Jack (pseudonym) was referred to our OA CMHT from his GP. He was temporarily living with his brother Mo, having previously lived with his late mother and been her main carer. Jack had struggled with psychotic experiences including voice hearing and delusional thinking for many years, with his symptoms managed through anti-psychotic medication. Prior to our input, Jack had never been offered psychological therapy. During the previous year he began to experience an exacerbation of his symptoms. After an assessment with the CMHT, he was referred to Psychological Therapies while awaiting care-coordinator allocation.

Assessment in the CMHT had raised concerns about a possible Alzheimer’s diagnosis due to a low score on the Montreal Cognitive Assessment. However, upon our assessment, no memory difficulties were evident nor did Jack report any recent changes in his memory, cognition, or adaptive functioning. Further cognitive assessment was therefore not carried out. In our assessment, both Jack and Mo raised the possibility of Jack being neurodivergent and having a learning difficulty, although this had not been identified in his childhood. They reported a long history of Jack struggling with social interaction long before he had any psychotic experiences, as well as difficulty with abstracting and generalizing information, both of which can be characteristic of autism (American Psychiatric Association, 2013). They also noted he had always taken considerable time to process information, often needing things to be phrased more simply and clearly. Assessment of a possible autism spectrum condition was unlikely to be possible in the absence of an accurate neurodevelopmental history and was outside the scope of the current intervention and clinical need. Moreover, his difficulties were not severe enough to warrant being treated within a specialist intellectual disability service. However, it was considered that these learning difficulties, potentially in addition to his high levels of paranoia and anxiety, may have been what led to the question of a dementia being raised. These hypotheses, including the potential for identifying cognitive change or decline, were held as part of the formulation and the intervention was adapted as required.

Jack gave informed consent to be seen by a trainee clinical psychologist, and both Mo and Jack gave consent to record sessions, and for this case to be published and included in an anonymized report written for the clinician’s university. Although Jack reported often feeling unhappy, he at no point expressed any thoughts of life not being worth living or wanting to hurt himself or others. Jack reported no physical health conditions or concerns. His psychiatric medication included sertraline and risperidone 500mcg.

## Assessment

The authors gathered assessment information from past clinical notes and through two sessions with Jack. At assessment Jack described severe anxiety and worry regarding being arrested, which he was certain was due to happen imminently. He spoke about some work he had done for a contractor several years ago and reported worries about being called to trial for tax evasion. He was convinced that although he was innocent of any crime, the police would be able to imprison him. He described hearing a number of nasty voices linked to these worries, including a policewoman who would say she was going to arrest Jack, put him in jail, then ensure he would be homeless and bankrupt upon release, and this woman’s husband, also a police officer, who would threaten to beat him up.

Jack described himself as a ‘natural born worrier’. He felt a lot of responsibility as a child after losing his father at a young age and needing to help care for Mo, his younger brother. The exacerbation of his worries and development of psychotic experiences occurred after some challenging life events, including being defrauded by an employee of his bank in 2005, for which Jack blamed himself, and experiencing bullying by a previous supervisor at work who was both physically and verbally aggressive to him. Mo provided corroboration and further detail on these incidents, which he believed had triggered Jack’s current delusion. Given evidence that the content of delusions is often based on real past experiences of harm or victimization (Freeman, 2016) this seemed a plausible hypothesis.

To manage his worries Jack typically remained at home, always checking around for police if he did go out. He also avoided talking to anyone apart from Mo, for fear of people reporting him to the police. Jack struggled to sleep at night due to preoccupation with worry, often napping during the day as a result. Day-to-day, Jack spent time watching TV and sitting in the garden. Despite describing himself as a ‘natural born worrier’, Jack’s worries did not appear to generalize to anything other than his concerns about the police.

## Goals

Jack described wanting to be able to worry less and to feel safer when out and about. Although he described finding it very challenging to meet new people, he felt he would like to try a new hobby such as a woodworking course if he were able to escape his worries. We discussed whether attending a local Men’s Sheds group (a community group where older men come together to share and learn new skills) could be a useful goal to set, and Jack agreed this would be suitable to work towards.

## Outcome Measures

Table 1 displays the outcome measures and time points they were completed.

**Table 1:** Outcome Measures

Construct	Measure	When completed
Worry		
Dunn Worry Questionnaire (DWQ)	The DWQ (Freeman et al., 2020) is a ten item measure of general worry developed as an improvement to the Penn State Worry Questionnaire (Meyer et al., 1990). Scores range from zero to 40, with higher scores reflecting higher levels of worry. A score of 21 and above indicates clinically significant levels of worry.	Once at baseline (Phase A) and once at end of treatment (Phase B)
Visual analogue scales (VAS)	Two VAS were completed: How worried have you been about other people this week on a scale of 0 (not worried at all) to 10 (worried all the time)?’, and ‘How distressed have you been about your worries about other people this week from 0 (not worried at all) to 10 (worried all the time)?’. These scales are recommended for weekly use when using the Worry Intervention.	At the start of each intervention session (i.e. throughout Phase B)
Paranoia		
Revised Green et al Paranoid Thoughts Scale (R-GPS)	The R-GPTS-B (Freeman, Lister, et al., 2019) comprises eight items measuring ideas of persecution and shows excellent psychometric properties. Scores range from zero to 40, with scores of above 11 reflecting clinically significant paranoia, and scores of 18-27 the likely presence of a persecutory delusion. Although the measure has not been specifically validated for use with older adults, the measure does show invariance between age groups.	Once at baseline (Phase A) and once at end of treatment (Phase B)
Visual analogue scale (VAS)	An analogue scale ranging from 0 (don’t believe it at all) to 100% (believe it totally) was administered to measure conviction in Jack’ persecutory delusion that he would be unjustly arrested for tax evasion. This is a scale within the Psychotic Symptoms Rating Scale (PSYRATS) that is commonly been used as an outcome measure of delusional conviction (e.g. Freeman, Lister, et al., 2019)	Once at baseline (Phase A) and once at end of treatment (Phase B)
Wellbeing and functioning		
Clinical Outcomes in Routine Evaluation-Outcome Measure (CORE-10)	The CORE-10 (Barkham et al., 2005) measures wellbeing, functioning, problems/symptoms, and risk. The measure was initially developed for use in adult services, but has been validated for use in older adult populations (Barkham et al., 2005). Scores range from zero to 40, with higher scores depicting more severe difficulties. Presence of clinically significant symptoms (caseness) is defined as a score of 10 or above (Barkham et al., 2005).	Every week throughout baseline (Phase A) and intervention (Phase B)
Work and Social Adjustment Scale (WSAS)	The WSAS measures the impact of mental health difficulties on day-to-day functioning (Mundt et al., 2002). The scale has five items covering work, home management, social leisure, private leisure, and relationships. Scores range from 0 to 40 with higher scores indicating greater impairment. The scale demonstrates good internal consistency, reliability, convergent and criterion validity. Caseness is defined as a score of 10 or above (IAPT, 2011). This measure is used routinely in older adult services, although no known validation of the scale within older adult populations has been reported.	Every week throughout baseline (Phase A) and intervention (Phase B)

## Design

An AB design was followed. A three-week baseline period (A) was established before and during assessment, prior to intervention. The intervention phase (B) comprised weekly CBT sessions with measures completed at the start of each session. This design allowed inferences to be made regarding the impact of therapy on specified outcomes. Jack found completing questionnaires each week challenging and the assistance he needed to complete them could take considerable session time. Therefore, only service compulsory measures and worry analogue scales were completed weekly, with other measures completed just once at the start and end of treatment.

## Cognitive Behavioural Formulation

A shared understanding of Jack’s difficulties was built using Freeman’s cognitive model of paranoia (Freeman, 2016). This uses a “vicious flower” formulation to understand why paranoia is maintained. One of the key mechanisms within this model is worry, hence why this model was chosen. Two mini cycles that were to be the focus of the intervention were discussed and drawn out together with Jack (Appendix A, Supplementary Materials), and the clinician also developed a separate more complete formulation (Appendix B, Supplementary Materials). The first mini cycle shows how Jack’s worried thoughts made him feel anxious in his body, e.g., his heart would race. Consequently, he worried more, taking the anxiety to be a sign of something being wrong. The second mini cycle shows how Jack’s feelings of unsafety led him to worry, in turn making him feel even more unsafe, because his worries always focused on worst case scenarios. Jack also noted his worry meant he slept badly, did limited meaningful activity, and avoided engaging with others meaning his social network was small. These factors were included in the wider formulation.

A number of other variables were discussed with Jack that were thought to contribute to his feeling unsafe that also form part of the cognitive model of paranoia and were added to the clinician’s formulation. For instance, Jack’s experience of hearing nasty voices understandably made him feel unsafe. Jack also described some negative beliefs about himself that the clinician considered as important developmental factors in Jack’s presentation. He wondered whether he had a ‘weak mind’, possibly due to stigmatising cohort beliefs about psychosis held among some older adults (Farrer et al., 2008). Jack also reported often feeling different to others – not uncommon among older adults with neurodiversity and who struggle with social interaction (Hickey et al., 2018) and possibly exacerbated by his experience of bullying – a common feeling of self-vulnerability that paranoia can build upon (Freeman, 2016). Moreover, in the past year Jack had moved from being a carer for his mother for which he felt much pride, to being cared for by his younger brother. This transition in role investments, a concept within Laidlaw’s formulation for older adults (Laidlaw et al., 2003), may have contributed to Jack viewing himself more negatively.

To manage his worries about being unsafe Jack used safety behaviours of avoiding places where he felt the police might be more likely to catch him and checking around for police cars. Additionally, Jack showed evidence of sometimes jumping to conclusions when considering evidence for his beliefs, often struggling to consider alternative explanations. For example, upon hearing a siren he would tend to assume that the police must be coming to arrest him, and not consider alternative explanations.

Based on this formulation, testable hypotheses were developed.

1. The worry intervention will reduce Jack’s levels of worry as measured by the DWQ and weekly VAS.
2. Given worry is a maintenance factor for paranoia (Freeman et al., 2015), Jack’s paranoia as measured by the R-GPTS-B and delusional conviction will also reduce.
3. Improving Jack’s worry will allow him to engage in more meaningful daily activity and experience better wellbeing, evidenced by improved scores on the CORE-10 and WSAS.

## Intervention

Treatment comprised ten 60–75-minute sessions face to face over three months. Generic CBT for psychosis was considered as an option initially but given Jack’s high levels of distressing worry and the demonstrated effectiveness of worry intervention for reducing both worry and paranoia, it was decided a worry intervention would be tried initially. These options and the recommendation were explained to Jack in layperson terms, who agreed with and consented to the plan given reducing his worry was something he most wanted help with. The worry module of the Feeling Safe Programme was therefore followed. This intervention is typically six-eight sessions, but content was paced more slowly to account for Jack’s learning preferences. Frequent feedback was elicited to ensure sessions were clear, helpful, and well-paced, and short session summaries were written as Jack found these easier to review than the full intervention module booklets.

The intervention began with worry psychoeducation and a diary to identify Jack’s time spent worrying. This showed he spent up to 15 hours a day worrying and not engaged in any other form of activity. The diary also identified Jack’s triggers and ‘peak times’ of worry. Sitting in his living room unoccupied was a clear trigger, and peak times were first thing in the morning and last thing at night. To build motivation to reduce worry, in session two we identified Jack’s positive and negative beliefs about worry. Jack thought worrying helped him to organise his mind and prepare for bad things happening, but he also felt strongly that worrying made him feel distressed and anxious. Overall, therefore, Jack was strongly in favour of reducing his worrying.

The concepts of worry periods and worry postponement were therefore introduced in Session 3. These techniques aim to postpone worry until a designated time and place, allowing an outlet for worry that is time limited and controlled. Outside of worry periods the aim is to stay occupied with meaningful activity to help keep worry away. Given he spent so many hours each morning lying in bed worrying, Jack felt his worry period needed to be early in the day, else he would not be able to keep postponing his worry. He chose the location as a spare room he normally didn’t use. We began a list of enjoyable activities for Jack to engage in outside of worry periods. These included a puzzle book, history podcasts, and helping Mo prepare meals. Given the importance of structure as a tool for reducing boredom and inactivity among older adults (Baumann, 2013), to do lists and timetables were co-created with Jack to support him to increase his activity.

Jack initially found the concept of worry periods difficult. Given we were aiming to reduce worry, he felt allowing himself to worry at all would make it escalate and impossible to control. We tested this meta-worry in a behavioural experiment, where Jack compared his worry on days with and without worry periods. Although his beliefs about the worry becoming uncontrollable did not come true, Jack also did not find the worry periods helped him to worry less outside of the periods. He felt he did not actually need an outlet for his worries, with activity engagement being the most helpful tool for reducing worry. Eliminating worry periods is ultimately the desired outcome by the end of a patient’s recovery from worry and given Jack’s worry was already improving considerably, we agreed he would continue without using worry periods.

Three sessions then focussed on new exercises for letting go of worry. These included getting active, connecting with others, and using positive imagery. Jack practised these between sessions, with one task being to visit Men In Sheds, helping Jack to try to meet one of his goals for therapy. Finally, we ended with two review sessions where a therapy blueprint was created.

Jack engaged extremely well in therapy. With Jack’s agreement, Mo was present for the first six sessions so that he understood the treatment tools and tasks and could support Jack with them between sessions when required. Jack then attended sessions 7-9 alone so he could practise retaining and implementing the information without assistance, with Mo returning for the final review session.

Throughout, the clinician aimed to instil hope in Jack for successful recovery, keeping in mind the stigmatising cohort beliefs that many older adults today can hold about psychosis. Validation and empathy were given for how distressing Jack’s worries were, and curiosity was shown regarding the evidence for his delusional beliefs whenever Jack raised this. These elements helped to form a strong therapeutic relationship.

## Results

**Figure 1:**
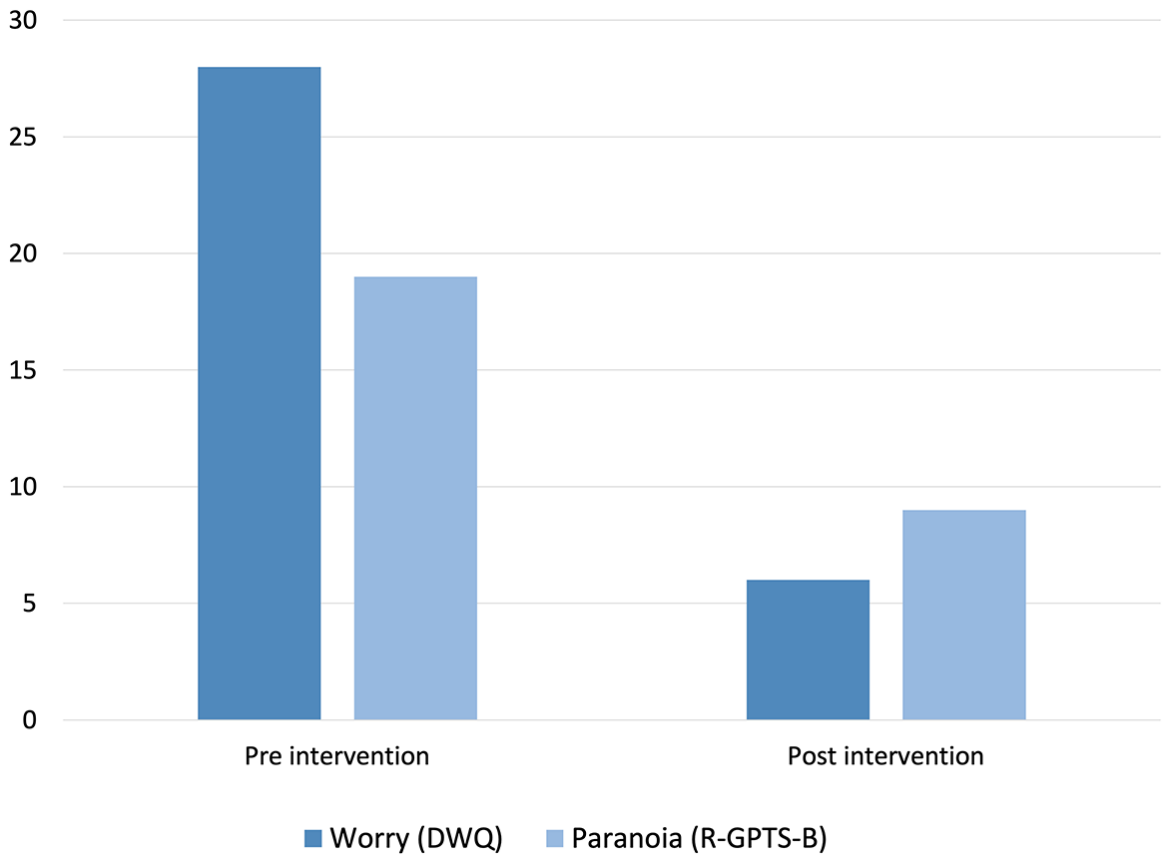
Pre-and-Post-Intervention Scores for Worry and Paranoia

**Figure 2:**
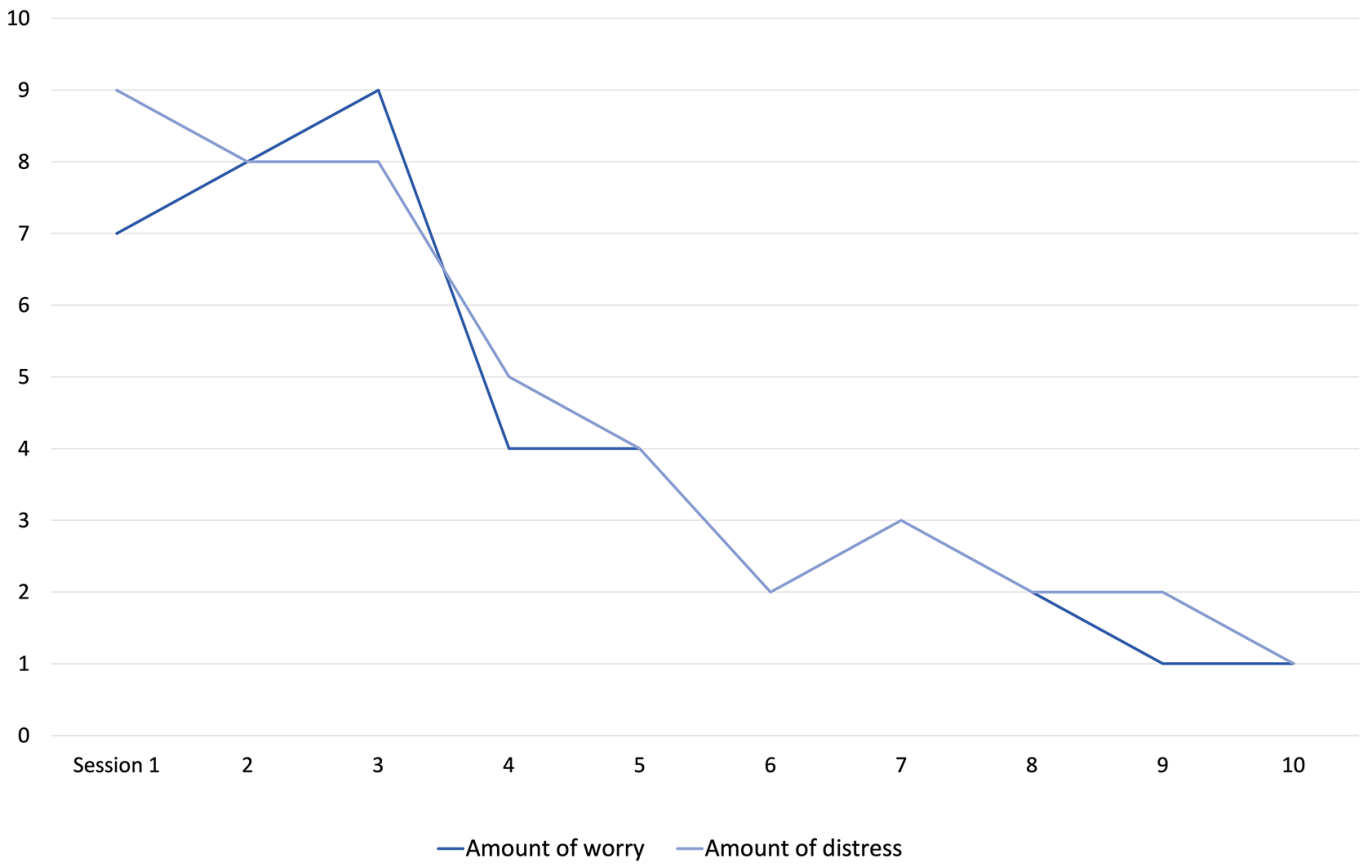
VAS Scores for Worry and Associated Distress During Phase B

Figure 1 shows Jack’s scores on the standardised worry and paranoia measures pre-and-post-treatment. Pre-intervention, Jack was experiencing clinically significant levels of worry (a score of 28, where above 21 discriminates clinical severity), and paranoia (a score of 19, where above 11 discriminates clinical severity). Post-intervention, Jack’s worry reduced hugely to a score of just 6, and his paranoia 9, both scores falling below clinical cut-offs. Scores on analogue scales of worry (Figure 2) also showed this, and an equally large drop was seen in Jack’s delusional conviction, which fell from 100% at baseline to 25% at end of intervention.

**Figure 3:**
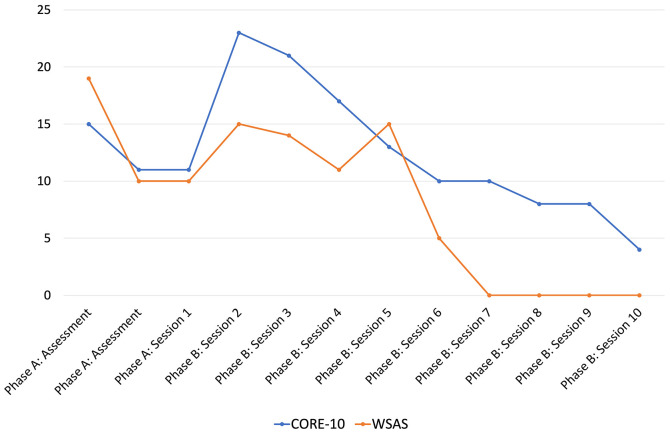
Changes in Wellbeing and Functioning Over Phases A and B

Figure 3 shows Jack’s scores on the CORE-10 and WSAS measures across baseline and intervention. Jack completed these before each session began, meaning the scores for Treatment Session 1 were still part of the Phase A. At first baseline, Jack’s scores on both measures were indicative of ‘moderate’ difficulties. Neither baseline remained entirely stable, however, with the scores reducing to the ‘mild range’ for the second and third baseline measures. Scores on both measures extended into the ‘moderate-severe’ range early on in treatment, gradually reducing until scores were very low (CORE-10) or indeed zero (WSAS) by end of treatment.

## Discussion

This report describes the use of a worry intervention in an older adult with a persecutory delusion. All three hypotheses were supported: By the end of treatment, Jack’s worry considerably reduced, as did his paranoia and delusional conviction, and his wellbeing and daily functioning improved, all to a clinically significant level. Although not formally measured, Jack also reported no longer hearing his nasty voices by the end of treatment.

Although there were large improvements overall, it was discussed in therapy why some of the measures initially increased in score (meaning a worsening of symptoms). At assessment, Jack was clear that he was worrying a lot and wanted to reduce this, but it was not until we began a worry diary that he realised just how much time each day he spent worrying and how much of an impact this was having on his daily life and wellbeing. He therefore reflected that his earlier scores had perhaps been an understatement of his difficulties.

Conversely, there was a striking reduction in Jack’s worry on the VAS after Session 3. This occurred after introducing the concepts of worry periods and worry postponement, and therefore when Jack began building more activity into his day. While this did not immediately translate into similar improvements on the wellbeing and daily functioning measures, this was perhaps due to how hard Jack found he had to work at postponing and reducing his worry. Alternatively, there may simply be a higher margin of error in Jack’s CORE and WSAS scores as compared to the VAS because Jack found these measures difficult to complete. Within the WSAS Jack struggled to separate out the extent to which he had completed daily tasks, with the extent to which worry had impacted his ability to complete daily tasks. It therefore took some time for the clinician to find accurate question phrasing that allowed Jack to understand what was being asked and therefore respond accurately.

There are a number of threats to validity in this report. The baseline period was brief, and to reduce the burden on Jack the non-routine measures were only completed once at baseline. Ideally, the R-GPTS and DWQ would have been measured throughout baseline, but given Jack required support to complete them this was not possible within available session time. Additionally, the WSAS was not stable between the first baseline measure and latter two, with a smaller but still notable lack of stability also evident in the CORE. As discussed, Jack initially found these measures confusing to complete. While the intervention could have been delayed, achieving a longer, more stable baseline, this was considered unethical given there was availability to see Jack immediately. These design limitations mean caution is warranted with interpretating the results; it is possible Jack could have experienced natural recovery without the intervention. However, the extent of improvement was significant, and tallied closely with different stages of intervention, which does support the conclusion that the intervention was the primary cause of improvement.

Post-intervention Jack reported feeling proud of his achievements and confident for the future. Some concern about being arrested remained, but he acknowledged he could not be helped directly with this, and what had been most helpful was reducing his time spent worrying about it. Jack was therefore discharged from the Psychology team and CMHT. Due to limited capacity the CMHT had not been able to offer care-coordination during therapy, and Jack now felt he no longer needed any.

Overall, this report shows a brief intervention on worry led to large reductions in paranoia in an individual with a persecutory delusion. This supports the cognitive model of paranoia, where worry is a contributory causal factor in paranoia’s maintenance that can be targeted therapeutically (Freeman, 2016). Moreover, the report is a first step to showing this intervention can successfully be applied with an older adult, including where there is possible neurodiversity. It will be useful to test this further, including with those in their 70s, 80s, and 90s.

### Therapist’s Reflections on the Case and its Clinical Implications

When discussing this case in a multi-disciplinary meeting, the therapist (PB) was met with surprise by some colleagues who had expected treatment to primarily focus on reality testing Jack’s delusion and helping him consider alternative explanations. The successful results of this single case provided further clear and helpful evidence to the team on the importance of intervening on factors around an individual’s delusion, rather than always focussing directly on the delusion itself.

Having Mo as a co-therapist was also extremely helpful in this case, which provided learning for our service in terms of making more use of family members. Mo was very well engaged in the sessions, and able to motivate and remind Jack of homework tasks during the weeks that he may otherwise have forgotten. As a trainee clinician only working two days a week in the service and thus limited in capacity to do check-in phone calls in between sessions, this was particularly useful. Given his longer experience of communicating with Jack, Mo was also sometimes able to rephrase questions or explanations in a way that was more understandable to Jack. However, it was clear that their relationship was very respectful, kind, and stable. Mo was careful not to speak or act on behalf of Jack, but equally Jack was able to look to Mo for support when needed. It was reflected in supervision how things might have been different had their relationship been more challenging, and how the therapist might have needed to step in more frequently to manage this, potentially sectioning of parts of session to be conducted with only Jack.

We are also aware that in some ways this case felt somewhat different to other older adult cases. Perhaps most notably, Jack was not struggling with any comorbid physical health difficulties, a variable which often needs a lot of attention in older adult work. While it would have been easy to treat Jack the same as we might treat an adult of working age, mid-way through therapy it was helpful to step back and apply a Laidlaw formulation to his case and consider the potential impact of role investments and cohort beliefs in particular, even though these were not explicitly discussed during therapy.

## Supplementary Materials

The Supplementary Materials contain the following items (for access see Brown & Crabtree, 2023):

- Appendix A: Two mini formulations created with Jack.
- Appendix B: An enhanced formulation created by the clinician to guide intervention but not shared with Jack.

BrownP.
CrabtreeA.
 (2023). Supplementary materials to "Worry intervention in an older adult with a persecutory delusion: A single case experimental design"
[Online appendices]. PsychOpen. 10.23668/psycharchives.13202
PMC1086363938356897

## Related Versions

The work was completed as part of the first author’s Doctorate in Clinical Psychology at the University of Oxford.

## References

[sp1_r1] Brown P. Crabtree A. (2023). Supplementary materials to "Worry intervention in an older adult with a persecutory delusion: A single case experimental design" [Online appendices]. PsychOpen. 10.23668/psycharchives.13202 PMC10863639 38356897

